# Feature Binding of Sequentially Presented Stimuli in Visual Working Memory

**DOI:** 10.3389/fpsyg.2020.00033

**Published:** 2020-02-05

**Authors:** Anuj Kumar Bharti, Sandeep Kumar Yadav, Snehlata Jaswal

**Affiliations:** ^1^Center for Biologically Inspired System Science, Indian Institute of Technology, Jodhpur, India; ^2^Department of Electrical Engineering, Indian Institute of Technology, Jodhpur, India; ^3^Department of Psychology, Chaudhary Charan Singh University, Meerut, India

**Keywords:** feature binding, simultaneous presentation, sequential presentation, locations, visual working memory

## Abstract

Feature binding is a process that creates an integrated representation of an object. A change detection task with four stimuli is used to study color-shape binding of sequentially presented stimuli. Given the immense importance of locations in feature binding, and noting the confound of location information with simultaneous presentation, we compared simultaneous and sequential presentations when locations remained the same from study to test and when they changed randomly. In Experiment 1, sequential presentation implied showing the stimuli one by one to gradually build up the study display. There were no differences between the two modes of presentation in this experiment, although performance was better with unchanged locations than random locations. Experiment 2 used a sequential presentation when one stimulus vanished as the next was presented. An interaction effect showed that performance was much better with unchanged locations than random locations with simultaneous presentation, whereas locations had no effect in the sequential presentation condition. Three subsequent experiments, with drastically reduced presentation time for the display in the simultaneous presentation condition (Experiment 3), with blank intervals inserted after every stimulus in the sequential presentation condition (Experiment 4), and with a mask given immediately after the study-display presentation (Experiment 5), showed results similar to Experiment 2. Thus, we surmise that locations are a factor only in simultaneous presentation, and not in sequential presentation, and the differences between the two conditions can be attributed to post-perceptual factors within visual working memory.

Feature binding is the process by which different characteristics, such as, orientation, size, shape, color, and location, are integrated to create an object. Binding is a necessary process for accurate perception of the world. Not only does it allow the separation of figure and ground, but also the differentiation of one object from another. Objects in the real world differ in space as well as time. Presumably, feature binding helps us differentiate objects not only when they are present together at the same time in our experience, but also when they are experienced at different times, say in a sequence. Our aim in this research is to explore the factors – whether distinct or the same – which operate in the binding of simultaneously and sequentially presented stimuli.

For testing binding in laboratory environments, a change detection task is often used. A change detection task presents a study display and a test display. Participants need to detect whether the test display is the same or different as compared to the study display. The task can be used to test changes in uni-dimensional or multi-dimensional stimuli. When testing feature binding, all the features in the test display are the same as the study display, but their combination changes on some trials, thus the task essentially becomes “swap detection.”

Most studies of feature binding, using the swap detection task, simultaneously present the stimuli in the study display. Nevertheless, as mentioned earlier, the differentiation of objects can be over space or time. Differentiation over space (on diverse locations) is inevitable with simultaneous presentation, and separation over time yields sequential presentation. Accordingly, the array of objects in the swap detection task can be presented simultaneously or sequentially.

Simultaneous presentation of multiple objects utilizes the powerful cue of location and allows configural encoding as shown by many studies of uni-feature objects (Jiang et al., 2000; Blalock and Clegg, 2010). The importance of location in binding has been emphasized by feature integration theory (Treisman and Gelade, 1980; Treisman and Sato, 1990; Treisman, 2006; Huang et al., 2007) as well as guided search model (Wolfe, 1994). Feature integration theory (Treisman and Gelade, 1980) suggested that binding is mediated by the links of separate features to a common location. Treisman and Sato (1990) proposed that a “master map” of locations exists in our brain. Attention selects all the features associated with a particular location, and works as glue to bind those features. Neuroscientists have found the evidence for such a master map. O’ Keefe and Nadel (1978) found the existence of place cells in the hippocampus. Hartley et al. (2007) supported the role of the hippocampus in topographical processing in short-term memory. Jacobs et al. (2013) did single-cell recordings from patients of epilepsy, which indicated grid cells in the entorhinal cortex and place cells in the hippocampal region. Recently, Koen et al. (2017) showed that the hippocampus plays a critical role in forming and maintaining complex bindings. Several studies have also shown that activity in the retinotopically organized sub-regions of the visual and parietal cortex is critical for visual short-term memory storage (reviewed in Xu, 2017). Behavioral studies show that location is remembered better than colors (e.g., Wheeler and Treisman, 2002). Studies also show that bindings are more vulnerable to location change and suggest that location plays a central role not only in encoding but also in maintenance and retrieval of bound objects (Treisman and Zhang, 2006; Hollingworth, 2007; Richard et al., 2008; Logie et al., 2011). Although Udale et al. (2018) provide recent evidence for strategic retrieval and decision-making by participants when task demands discourage the use of location cues, “in place” matching appears to be the default strategy of most participants even in their work. Thus, simultaneous presentation of multiple objects is considered crucial for binding by many researchers.

Nevertheless, some researchers have contrasted simultaneous and sequential modes of presentation in binding tasks. Allen et al. (2006) used a shape-color binding task with both modes of presentation. Results showed that performance was less accurate with sequential mode of presentation. Brown and Brockmole (2010) tested binding deficits in older and younger people using simultaneous and sequential modes of presentation. Although the results did not show any effect of age on binding, performance was worse with sequential presentation for both groups.

Other research groups showed that sequential presentation is better than simultaneous presentation. Fougnie and Marois (2009) used a visual working memory task in which participants had to detect changes in color, shape, either color or shape, and binding. During the retention interval, they performed a multiple object-tracking task. Results suggested that impairment caused by the secondary task was significantly reduced when objects were shown sequentially at the center of the screen. A comparison of the results of their separate experiments with simultaneous and sequential presentations shows slightly better baseline performance by participants in the sequential condition. Yamamoto and Shelton (2009) used real-life scenarios and found that sequential presentation of objects makes it easy to memorize them. They used a room layout and six different objects. Participants were shown these objects either simultaneously for 30 s or sequentially for 2.5 s per object, with the whole array being shown twice. Results showed better performance with sequential presentation. Ihssen et al. (2010) have also shown the superiority of sequential presentation. Their experiment had three conditions. In simultaneous presentation, they showed eight objects at the same time for 700 ms. In the sequential mode, they showed two displays sequentially, containing four objects at a time for 350 ms. In the third condition, the eight objects were repeated (shown twice), with each display shown for 350 ms. Results showed better memory performance in the sequential and the repeated modes.

Thus, conflicting results for different modes of presentation are observed in research studies. Simultaneous presentation increases the competition among stimuli, and the errors from within the memory set are more common than when stimuli are presented sequentially. Emrich and Ferber (2012) used eight colored squares presented either simultaneously for 400 ms, or divided into two displays of four squares each, presented sequentially. Error in detection of a particular color was more with simultaneous presentation. Haskell and Anderson (2016) also found reduced error variance with sequential rather than simultaneous presentation of circular gratings requiring judgments of orientation. Reduced errors with sequential presentation could be associated with better or equivalent performance (to simultaneous presentation), obtained at times with sequential presentation, especially in real-life conditions, where experience or familiarity with stimuli might mitigate the effects of competition and increase the distinctiveness of stimuli.

However, in experimental tasks used in the laboratories, simultaneous presentation generally yields better performance. Perhaps, this is because it allows configural encoding of the rather simple stimuli used in laboratory experiments. Stimuli can be encoded and remembered in relation to each other and form a visual pattern more easily when presented simultaneously than when presented sequentially. The relative location of stimuli is a powerful cue in simultaneous presentations. If we really wish to compare simultaneous presentation with sequential presentation, confound of location with simultaneous presentation must be removed/controlled. This is particularly important in binding studies, given the immense importance of locations in binding.

Some researchers have used a single probe at test to negate the role of location. Other researchers have attempted to control the effect of location by presenting stimuli at unchanged locations. However, because other features may be addressed through a “location map,” presenting single probes or the test stimuli at unchanged locations is not an adequate control. The huge literature on classical conditioning shows that to really break a link, it is important to randomly associate the elements participating in the link (Rescorla, 1967). To make locations irrelevant, the best strategy is to randomize them from study to test.

Thus, to unravel the effects of mode of presentation and relative locations, it seems imperative to orthogonally manipulate these two variables. In the present experiments, simultaneous and sequential presentations are compared when stimuli are presented in unchanged locations and when they are presented in random locations.

Some recent experiments studying the effect of mode of presentation on bindings with locations controlled in different ways are relevant here. Gorgoraptis et al. (2011) found that sequential presentation leads to low memory precision and more misbindings. They tested the binding of color and orientation with both modes of presentation. In the study display, they presented a number of colored bars with different orientations. In response, participants needed to adjust the orientation of the probed colored bar. The test bar was always shown at fixation. Locations were randomized in the study display in each trial in sequential as well as simultaneous presentation modes. But, in the design of this study, location was only randomized as a controlled variable, it was not an independent variable to enable an assessment of its effect in the experimental results. In another study, Pertzov and Husain (2014) using sequential presentations only compared performance in same and different locations, showing the advantage of same locations. But, in this experiment also, mode of presentation and location were not completely crossed.

Jaswal and Logie (2011) studied simultaneous and sequential modes of presentation in separate experiments keeping locations constant in one condition and randomizing locations from study to test in the other condition. Performance was inferior with sequential presentation when the participants never saw all the stimuli together in the test display, even when locations of the stimuli remained unchanged. This suggests that simultaneous presentation is better, because it gains from the relative location information concomitant with simultaneous presentation. In fact, when location was randomized, and thus rendered irrelevant to the task, there was no significant difference in performance between the simultaneous and sequential presentation experiments. Nevertheless, simultaneous and sequential presentation modes were not directly compared in their experiments and the set size at six was well beyond visual working memory capacity. Our experiments remedy this shortcoming and compare simultaneous and sequential presentations in the same experiments with set size four.

In conclusion, behavioral studies have shown equivocal results regarding performance with simultaneous and sequential presentation. In most behavioral experiments, simultaneous presentation is confounded with location information that either encourages configural encoding (leading to better performance) or increases competition and misbinding (leading to decrement in performance). An important strategy for extricating the effects of mode of presentation and location is to manipulate both of them as separate independent variables. This is what we have done in our experiments. Five experiments are being reported here. In every experiment, simultaneous and sequential presentation modes are fully crossed with unchanged and randomized locations in a 2 × 2 design. The specific aims, design, and the results of each experiment are described in the next sections.

## Participants

A random and independent sample of 18 participants was selected for each experiment. All experiments use a repeated measures design with both factors being within subjects. *A priori* analyses of such a design is not supported by programs such as G*Power which estimate sample sizes. Thus, the sample size was decided on the basis of similar experiments reported in Jaswal and Logie (2011), although these experiments never compared simultaneous and sequential presentations together. They used 12 participants, so we decided to use more participants than their experiments, and recruited 18 participants in each experiment. It is pertinent here to mention that repeated measures designs are more powerful than independent samples designs. Thus, there were 90 participants in all five experiments. All participants were male undergraduates in the age range 18–22 years, reported normal or corrected to normal visual accuracy, and were paid a nominal amount as honorarium. Informed consent was taken from all participants after explaining the task, but without revealing the hypotheses.

## Apparatus and Stimuli

All experiments were designed in E Prime 2.0 (Psychology Software Tools, 2008) and were conducted on a Sony Vaio laptop with a 14 inch screen placed at a distance of about 70 cm from the participant. The screen had 100% brightness with a resolution of 1,366 × 768 pixels and an Intel HD Graphics card. The four stimuli in each display were random combinations of four shapes (diamond, ring, triangle, and plus) and four colors (red, green, yellow, and blue). All stimuli were made in a square frame (110 × 110 pixels) creating a visual angle of approximately 2.05° × 2.05° and were presented on a gray screen in a 3 × 4 invisible grid of 338 × 448 pixels such that they remained in foveal vision, subtending a visual angle of approximately 6.28° × 8.30°.

## Experiment 1

The experiment aimed to study the effect of mode of presentation and locations on feature binding, using a change detection paradigm. We compared simultaneous and sequential presentations as the two levels of mode of presentation, and unchanged and randomized locations as the two levels of locations. As we aimed to unravel the confound between simultaneous presentation and locations, keeping these two factors as the two independent variables seemed to be a good starting point.

**Figure 1:**
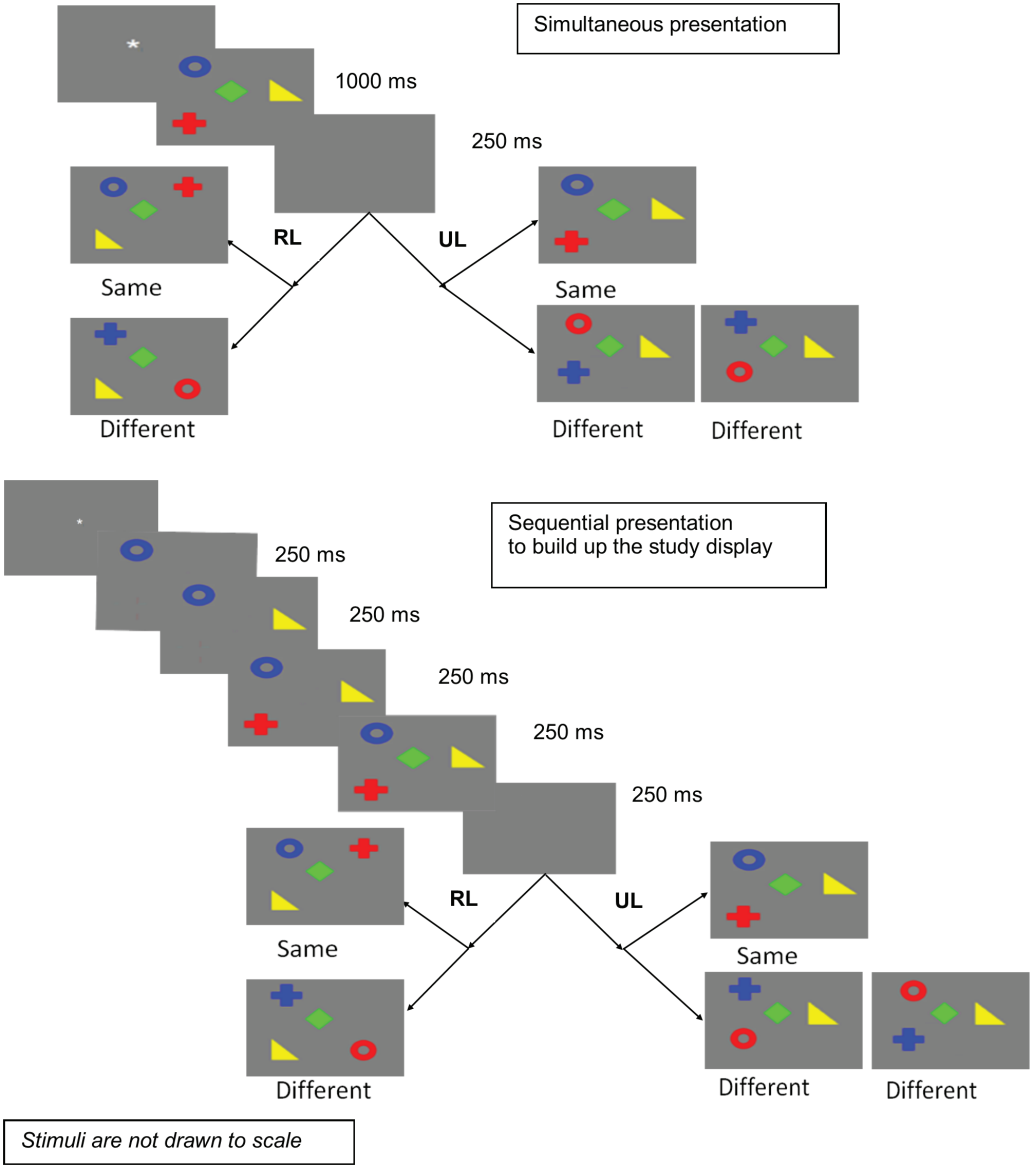
Simultaneous and sequential presentations in Experiment 1 (RL, random locations; UL, unchanged locations).

In this experiment, the sequential condition involves presenting the stimuli one by one, to build up the study display, as shown in Figure 1. Thus, in the sequential condition, an additional (temporal) cue is present. This might enhance performance in the sequential presentation condition relative to the simultaneous presentation condition. On the other hand, performance might be reduced in the sequential presentation condition relative to simultaneous presentation, if presenting stimuli one by one hampers configural encoding.

Further, unchanged locations from study to test are expected to yield better performance as compared to randomized locations, given the importance of locations as a cue in feature binding.

### Design and Procedure

The experiment was designed as a 2 × 2 factorial experiment with repeated measures on both factors. The two independent variables were mode of presentation (simultaneous vs. sequential) and locations (unchanged vs. random). The trials for unchanged and random locations were mixed randomly within each block of simultaneous and sequential presentations, which were counterbalanced across participants. On half the trials comprising the unchanged locations condition, the stimuli appeared in the same locations as the study display. On the other half of the trials, comprising the random locations condition, the locations of stimuli in the test display were randomized from the study display to the test display. Figure 1 illustrates the design and procedure in each trial.

Each trial started with a fixation display. When the participants were ready, they pressed any key to move to the study display. The study display comprised four stimuli, which were random combinations of four colors and four shapes in each trial. The participant was to remember the bindings between colors and shapes. Simultaneous presentation implied all four stimuli presented at the same time in a single display. For sequential presentation, stimuli were presented one by one such that the display was gradually built up. Previous stimuli remained on screen as the next appeared. The study display remained on the screen for 1,000 ms for simultaneous presentation. In the sequential presentation condition, starting from the first stimulus, each next stimulus appeared after 250 ms, with all four on screen only for the last 250 ms out of a total of 1,000 ms. Thus, the total exposure duration for both presentation modes was the same. Thereafter, a blank interval was introduced for 250 ms and then a test display appeared with four stimuli. The task of the participant was to detect if any of the four stimuli changed in the binding of color and shape from the study display to the test display in each trial. The binding change happened only on 50% trials in each condition. When the change occurred, it was actually a swap between any two stimuli. Note that the participants cannot do the swap detection task if they remembered the colors alone or shapes alone, as all the colors, and all the shapes, were repeated in the test display. Whenever a swap occurred, half the time colors changed locations, and half the time, shapes changed locations. This is experienced as different only when locations are unchanged. In the randomized locations condition, the experience of the participants does not differ for color swaps or shape swaps. The participants pressed equally separated keys for “different” and “same” to record whether they were able to detect a change in binding in each trial.

The participant had to complete the experiment in a single session. Before commencing the experiment, each participant completed 24 practice trials for each block, i.e., 48 trials in all. The experiment was completed in two blocks of 192 trials each, 384 trials in all. There was an equal number of each trial type in each block for practice as well as experimental trials. Articulatory suppression was used in each trial. The participant had to say the word “the” repeatedly from the fixation screen until after the response was given.

### Results

**Figure 2:**
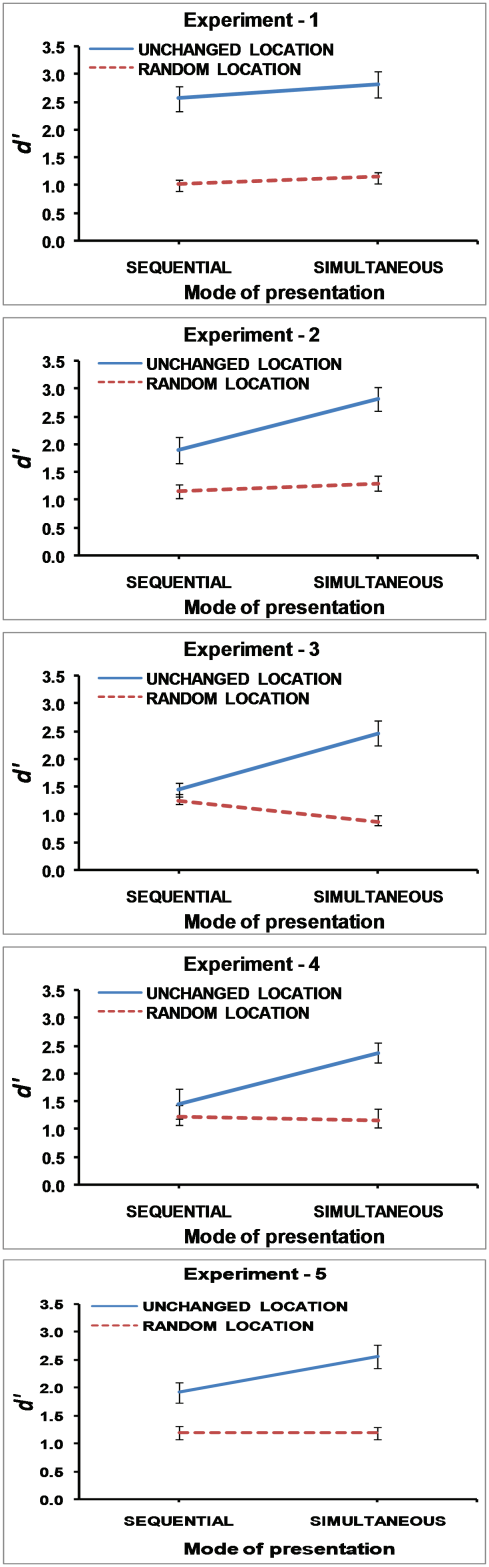
Mean d prime scores in Experiment 1, 2, 3, 4, and 5. The error bars represent ±1 Standard Error.

Mean change detection performance calculated from d primes is shown in Figure 2 for all experiments.

A repeated measures *ANOVA* revealed a significant main effect of unchanged and random locations, *F*(1,17) = 82.592, *MSE* = 0.559, *p* < 0.001, *partial η^2^* = 0.829, BF_10_ = 2.549 × 10^11^ such that overall performance was reduced when locations were randomly changed from study to test display than when locations were unchanged. Neither the main effect of mode of presentation, *F*(1,17) = 1.089, *MSE* = 0.609, *p* = 0.311, *partial η^2^* = 0.060, BF_01_ = 3.44, nor the interaction effect, was significant, *F*(1,17) = 0.140, *MSE* = 0.394, *p* = 0.713, *partial η^2^* = 0.008, BF_01_ = 3.230. The model comprising both the main effects and the interaction effect (BF_10_ = 3.464 × 10^10^) was compared with a model comprising only the main effects (BF_10_ = 1.119 × 10^11^). The model comprising only the main effects better fit the data by a factor of 3.23:1.

Table 1 shows the means of d prime scores in all experimental conditions in this and all other experiments. Table 2 shows the hits and Table 3 shows the false alarms in all experiments.

**Table 1:** Mean d prime scores in all experimental conditions in the five experiments.

	Sequential presentation						Simultaneous presentation					
	Unchanged locations		Random locations		Total		Unchanged locations		Random locations		Total	
	Mean	SD	Mean	SD	Mean	SD	Mean	SD	Mean	SD	Mean	SD
Experiment 1	2.568	0.962	1.021	0.357	1.794	1.076	2.815	1.009	1.158	0.511	1.986	1.168
Experiment 2	1.896	1.021	1.164	0.545	1.530	0.909	2.824	0.941	1.300	0.517	2.062	1.091
Experiment 3	1.450	0.501	1.257	0.501	1.354	0.517	2.466	0.966	0.875	0.319	1.670	1.088
Experiment 4	1.456	1.161	1.218	0.932	1.336	1.075	2.382	0.749	1.153	0.543	1.768	0.910
Experiment 5	1.913	0.774	1.198	0.463	1.556	0.741	2.556	0.923	1.189	0.520	1.873	1.029

**Table 2:** Hits in all experimental conditions in the five experiments.

	Sequential presentation						Simultaneous presentation					
	Unchanged locations		Random locations		Total		Unchanged locations		Random locations		Total	
	Mean	SD	Mean	SD	Mean	SD	Mean	SD	Mean	SD	Mean	SD
Experiment 1	0.761	0.029	0.577	0.024	0.669	0.146	0.759	0.028	0.588	0.023	0.673	0.138
Experiment 2	0.598	0.031	0.570	0.021	0.584	0.112	0.721	0.035	0.604	0.023	0.662	0.138
Experiment 3	0.598	0.023	0.614	0.029	0.606	0.111	0.706	0.033	0.600	0.027	0.653	0.137
Experiment 4	0.582	0.037	0.610	0.035	0.596	0.152	0.772	0.025	0.647	0.032	0.709	0.137
Experiment 5	0.631	0.020	0.627	0.023	0.629	0.093	0.714	0.023	0.650	0.026	0.683	0.110

**Table 3:** False alarms in all experimental conditions in the five experiments.

	Sequential presentation						Simultaneous presentation					
	Unchanged locations		Random locations		Total		Unchanged locations		Random locations		Total	
	Mean	SD	Mean	SD	Mean	SD	Mean	SD	Mean	SD	Mean	SD
Experiment 1	0.068	0.016	0.217	0.022	0.142	0.111	0.050	0.011	0.197	0.036	0.124	0.135
Experiment 2	0.122	0.030	0.209	0.041	0.166	0.157	0.046	0.014	0.175	0.026	0.111	0.111
Experiment 3	0.153	0.033	0.199	0.034	0.176	0.143	0.077	0.024	0.288	0.031	0.182	0.157
Experiment 4	0.199	0.044	0.240	0.038	0.219	0.176	0.090	0.025	0.254	0.040	0.172	0.163
Experiment 5	0.104	0.018	0.213	0.026	0.158	0.110	0.055	0.013	0.233	0.023	0.121	0.121

### Discussion

In accordance with earlier studies, (Jaswal and Logie, 2011; Logie et al., 2011), Figure 2 clearly shows that performance is better with unchanged locations than random locations. However, there is no significant difference between simultaneous and sequential presentation. Building up the study display by presenting stimuli one by one, and thus providing an additional temporal code, does not lead to any better performance than simultaneous presentation. This suggests that the difference between the two modes of presentation is not contingent on a temporal code alone. Perhaps other factors are more important in making simultaneous presentation better than sequential presentation. Alternatively, similar performance in the two modes of presentation may result because simultaneous presentation is also like sequential presentation as participants most likely encode even simultaneously presented stimuli one by one as suggested by eye-tracking studies (e.g., Becker and Rasmussen, 2008).

## Experiment 2

For sequential presentation in this experiment, stimuli were presented one by one such that the previous stimulus vanished as the next was presented. In such a sequential presentation, retention of the earlier stimuli becomes difficult because any given stimulus may overwrite the representation of the earlier stimuli. In the absence of previous stimuli, relational or configural encoding is much more difficult. Thus, this kind of presentation utilizes only a temporal cue in the absence of configural encoding. The performance of the participants is expected to be lesser with sequential presentation as compared to simultaneous presentation.

Further, because the representation of stimuli includes location as a feature and is thus a spatiotopic representation, feature swaps in the unchanged locations condition will be easier to detect than in the random locations condition. Also, since this spatiotopic representation is expected to exist more clearly with simultaneous presentation, therefore, the difference in performance between the unchanged and randomized locations conditions is likely to be more with simultaneous presentation rather than sequential presentation.

### Design and Procedure

**Figure 3:**
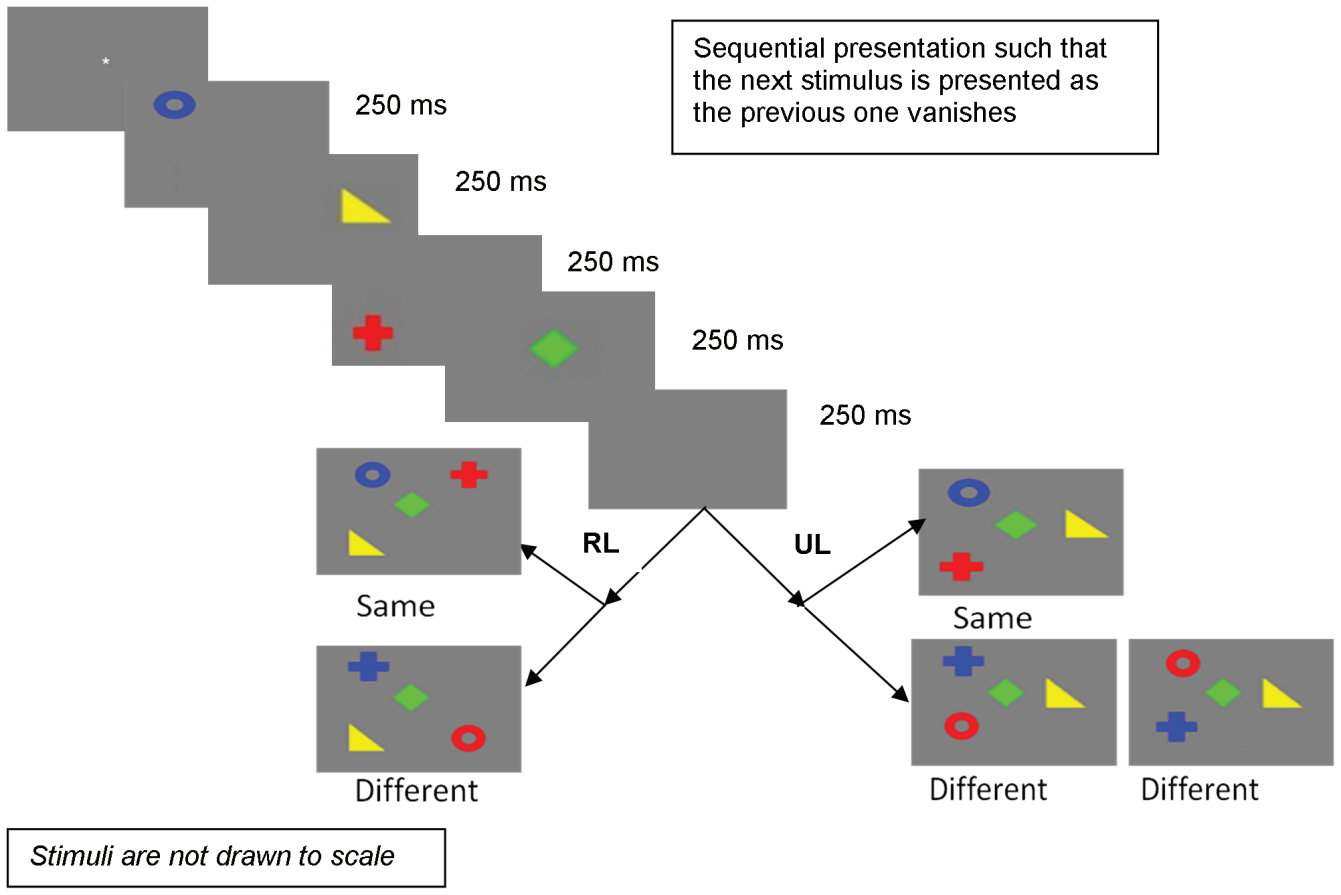
Sequential presentation in Experiment 2 (RL, random locations; UL, unchanged locations).

The design and procedure were the same as Experiment 1, except that sequential presentation involved presenting the stimuli one by one such that the previous stimulus vanished as the next stimulus was presented. Figure 3 depicts the procedure.

### Results

A repeated measures *ANOVA* revealed a significant main effect comparing unchanged and randomized locations, *F*(1,17) = 34.587, *MSE* = 0.662, *p* < 0.001, *partial η^2^ =* 0.670, BF_10_ = 6.939 × 10^5^. Overall performance was reduced when locations were randomly changed from study to test display than when locations were unchanged. The main effect comparing simultaneous and sequential presentations was also significant, *F*(1,17) = 15.609, *MSE* = 0.327, *p* < 0.001, *partial η^2^* = 0.479, BF_10_ = 3.245, with performance being better with simultaneous presentation than sequential presentation of stimuli. The interaction between mode of presentation and locations, *F*(1,17) = 10.370, *MSE* = 0.272, *p* < 0.005, *partial η^2^* = 0.379, BF_10_ = 4.378, was also significant. Figure 2, which shows the mean change detection performance calculated from d primes, substantiates that the difference in performance between unchanged locations and randomized locations is much greater with simultaneous presentation, *t*(17) = 6.608, *p* < 0.001, *d* = 1.577, BF_10_ = 4.607 × 10^3^, than with sequential presentation, *t*(17) = 3.254, *p* < 0.005, *d* = 0.767 BF_10_ = 9.994.

To compare the results of Experiment 1 and 2, three-way analysis of variance was carried out, taking experiments as a between-subjects factor, and mode of presentation and locations as the two repeated measures factors. The main effect of experiments was not significant. However, the interaction of experiments with location, *F*(1,34) = 3.311, *MSE* = 0.611, *p* < 0.078, *partial η^2^* = 0.089, BF_10_ = 1.404, and the three-way interaction, *F*(1,34) = 3.136, *MSE* = 0.333, *p* < 0.086, *partial η^2^* = 0.084, BF_01_ = 1.127, trend toward significance. The three-way interaction was assessed by comparing the model comprising the three-way interaction and all possible main and two-way interaction effects (BF_10_ = 1.062 × 10^18^) with a model comprising all three main effects and the three possible two-way interaction effects (BF_10_ = 1.197 × 10^18^). The data fit better with a model without the three-way interaction only by a factor of 1.127:1. This ratio being quite low, and the *p* < 0.084 of the three-way interaction trending toward significance, we infer that the performance of participants is different in the two experiments.

### Discussion

The sequential presentation in this experiment presents a stimulus as the previous one vanishes. This provides a temporal cue, but does not allow configural encoding. Thus, we find that performance is not only better with simultaneous presentation, but also that within this condition, performance is better with unchanged locations, because it is in this condition that maximum advantage can be derived from configural encoding aided by the feature of locations.

Mode of presentation has a significant effect only in Experiment 2, not in Experiment 1. This implies that location is a more advantageous cue than temporal presentation for feature binding. The experiment clearly revealed the advantage of configural encoding with the aid of location information for simultaneous presentation of stimuli. The temporal cue alone is not sufficient for feature binding in the visual domain.

Since it is only in Experiment 2 that mode of presentation showed a significant difference, the further reported experiments also used sequential presentation with the previous stimulus vanishing as the next one is presented.

## Experiment 3

One of the reasons for simultaneous presentation yielding better performance than sequential presentation in Experiment 2 could be its presentation time, i.e., 1,000 ms. This presentation time was kept at 1000 ms in Experiment 2 to equate it with the total presentation time of the sequential presentation, where each of the four stimuli was presented for 250 ms. In Experiment 3, we reduced the presentation time of the study display in the simultaneous presentation condition to 250 ms to make it equal to *one* stimulus of sequential display. Thus, one can say that participants were tested at the other logical extreme, as far as presentation time was concerned. Longer presentation time generally leads to better performance, although there are thresholds for liftoff of performance as well as when it reaches an asymptote (Busey and Loftus, 1994; Loftus and McLean, 1999). The time-based resource-sharing model of working memory (Barrouillet et al., 2004; Barrouillet and Camos, 2007) suggests that increasing the study display duration should improve performance for it allows more time for encoding and processing of stimuli. Pashler (1988) reported a significant but small increase in memory for 10 consonants presented simultaneously for 100, 300, and 500 ms. Liu and Jiang (2005) asked participants to remember objects in scene images to find that 250 ms allowed only about one object to be retained in memory. If the superior performance in simultaneous condition is indeed due to the long presentation time, reducing the presentation time of the study display in this way should drastically reduce performance in the simultaneous presentation condition, rendering it lesser than or no different from performance under the sequential presentation condition.

### Design and Procedure

The design and procedure were the same as Experiment 2 except that the study display in the simultaneous presentation condition was shown only for 250 ms.

### Results

Mean change detection scores calculated from d primes are shown in Figure 2. A repeated measures *ANOVA* revealed the main effect of unchanged and randomized locations, *F*(1,17) = 60.598, *MSE* = 0.237, *p* < 0.001, *partial η^2^* = 0.781, BF_10_ = 3.984 × 10^4^, in that overall performance was reduced when locations were randomly changed from study to test display than when locations were unchanged. The main effect comparing simultaneous and sequential presentations was also significant, *F*(1,17) = 7.459, *MSE* = 0.242, *p <* 0.014, *partial η^2^* = 0.305, BF_01_ = 1.226, with performance being better with simultaneous than sequential presentation. The interaction effect was also significant, *F*(1,17) = 23.061, *MSE* = 0.381, *p* < 0.001, *partial η^2^* = 0.576, BF_10_ = 1.760 × 10^4^. As depicted in Figure 2, there is a significant difference between unchanged and randomized locations with simultaneous presentation, *t*(17) = 7.137 *p* < 0.001, *d* = 1.682, BF_10_ = 1.121 × 10^4^, but the difference is not significant for sequential presentation, *t*(17) = 1.406, *p* = 0.178, *d* = 0.331, BF_01_ = 1.773.

A comparison of Experiment 2 and 3 by using a three-way *ANOVA* showed that neither the main effect of experiments nor any of its interactions were significant. Bayes factors were computed for each combination of main and interaction effects. A model comprising the three-way interaction with all the three main and interaction effects (BF_10_ = 5.608 × 10^16^) was compared with a model of three possible main and interaction effects without the three-way interaction effect (BF_10_ = 7.173 × 10^16^). The model with a three-way interaction was a slightly better fit for the data by a factor of 1.27:1.

### Discussion

The pattern of results obtained in this experiment is the same as that obtained in Experiment 2. Reducing the presentation time of the simultaneous display to a quarter of what it was in Experiment 2 had no effect on the performance of participants. Shorter exposure to the stimuli does not decrease (or increase) the performance of the participants, there being simply no significant difference between Experiment 2 and 3. These results indicate that the presentation time of the study display is not an important factor in the performance of the participants. Nevertheless, note that this experiment made changes only to the simultaneous presentation condition.

## Experiment 4

Although it seems that better performance under simultaneous presentation condition is obtained regardless of presentation time, one may argue that it is the time given for encoding the stimulus in the sequential condition, which is not enough. Ricker and Cowan (2014) tested forgetting in working memory as a function of time. They formulated the experiment comparing simultaneous and sequential conditions such that a blank interval is introduced between the stimuli in the sequential mode. Presumably, this helped in proper encoding of a stimulus, and it made performance in the sequential condition better than the performance in the simultaneous condition. Although they had tested memory for single features, analogously, we inserted blank intervals after each stimulus in the sequential presentation condition in Experiment 4, with a view to improving performance in this condition. We reasoned that blank intervals would aid consolidation or at least protect each stimulus from being overwritten by subsequent stimuli, and hence improve performance in the sequential condition.

### Design and Procedure

The design and procedure are the same as in Experiment 2 (depicted in Figure 3), except two related changes. In this experiment, a blank interval of 250 ms was introduced after each stimulus in the sequential presentation condition. Thus, the total time for sequential presentation becomes 1,750 ms, with four stimuli presented for 250 ms each and three blank intervals of 250 ms between the stimuli. The second change was an increase in display time for simultaneous presentation to 1,750 ms, to equalize it with the presentation time for sequential presentation. Experiment 3 (and its comparison with Experiment 2) had already shown that increasing the exposure duration has little effect on performance in the simultaneous condition. Also, a close study of Rhodes et al. (2016) showed that increasing presentation time from 900 to 2,500 ms yielded no significant difference in the retention of their participants for simultaneously presented stimuli.

### Results

Mean change detection performance calculated from d primes is shown in the Figure 2. A repeated measures *ANOVA* revealed the main effect of unchanged and randomized locations, *F*(1,17) = 31.006, *MSE* = 0.313 *p* < 0.001, *partial η^2^* = 0.646, BF_10_ = 72.278, in that overall performance was reduced when locations were randomly changed from study to test display than when locations were unchanged. The main effect of simultaneous and sequential presentations is not significant, *F*(1,17) = 3.096, *MSE* = 1.085, *p =* 0.096, *partial η^2^* = 0.154, BF_10_ = 1.47. Nevertheless, the interaction effect was significant, *F*(1,17) = 11.826, *MSE* = 0.372, *p* < 0.003, *partial η^2^* = 0.410, BF_10_ = 6.027. Figure 2 clearly depicts that the differential effect of unchanged and randomized locations is significant in the simultaneous presentation condition, *t*(17) = 8.438, *p* < 0.001, *d* = 1.989, BF_10_ = 8.765 × 10^4^, but not in the sequential presentation condition, *t*(17) = 1.026, *p* = 0.319, *d =* 0.242, BF_01_ = 2.617.

A comparison of Experiment 2 and 4 through a three-way *ANOVA* showed that neither the main effect of experiments nor any of its interactions were significant. Bayes factors were computed for all the combinations of main and interaction effects. To observe the three-way interaction effect, a model comprising the three-way interaction effect along with all the main and two-way interaction effects (BF_10_ = 2.587 × 10^10^) was compared with a model of all main and two-way interaction effects only (BF_10_ = 7.445 × 10^10^). The data fit better with the model without the three-way interaction effect by a factor of 2.87:1.

Another three-way *ANOVA* comparing Experiment 3 and 4 also did not show any differences between these experiments. Bayes factors were computed for all the possible combinations of main and interaction effects. The model comprising the three-way interaction and all the main and two-way interaction effects (BF_10_ = 2.897 × 10^10^) was compared with a model comprising only the main and two-way interaction effects (BF_10_ = 5.256 × 10^10^). The data fit better with the model without the three-way interaction by a factor of 1.818:1.

### Discussion

The main effect of locations and the interaction of locations and mode of presentation, both, are significant, as might be expected on the basis of the previous experiments. There is nothing new here. What is relatively more informative is that in this experiment, there is no significant difference between the two presentation modes. This might be because the overall performance in the simultaneous presentation condition decreased as compared with Experiment 2 (although the decrease does not lead to a significant main effect of experiments in the three-way *ANOVA*). The decrease in the performance of the participants in the simultaneous presentation condition with unchanged locations could be because the participants lost the iconic memory for the study display over the blank period. Alternatively, if the stimuli were already in the visual working memory, the participants could not sustain the relational encoding of the multiple stimuli in visual working memory. The next experiment will address whether and how far performance in this condition gains from iconic memory.

The performance of the participants in the sequential presentation condition remains the same as earlier experiments. Thus, it seems that the blank intervals, which yielded better performance with sequential presentation of uni-feature stimuli in the experiment by Ricker and Cowan (2014), conferred no advantage in our experiment to the multi-feature sequentially presented stimuli for feature binding. Blank intervals may protect uni-feature objects from decay and interference, but have no effect on bindings.

## Experiment 5

Better performance with simultaneous presentation of stimuli may also result due to iconic memory of the visual display for simultaneous presentation, affording the correct response more easily, especially in the unchanged location condition. Iconic memory preserves the stimulus pattern for some time after it has been presented, and then visual information is transferred to visual short-term memory. Masks of different kinds have often been used to wipe out iconic memory (e.g., Sperling, 1960; Neisser, 1967; Turvey, 1973; Becker et al., 2000). Studies by Phillips (1974); Loftus et al. (1985); Loftus et al. (1992) suggest that the icon does not persist beyond the initial 100–300 ms, and in fact, longer the stimulus presentation, shorter the duration for which the icon lasts (Coltheart, 1980).

Thus, to obliterate the effects of iconic memory from performance, we decided to use a visual noise mask for 250 ms immediately after the study display in all experimental conditions, and explore whether any changes would result in the pattern of performance. Particularly, we expected that if iconic memory is the reason why simultaneously presented stimuli are better retained with unchanged locations, performance in this condition would reduce as compared to Experiment 2. However, if the stimulus representations are already in visual working memory, then they would be immune to the mask and there will be no changes in the performance of the participants, as suggested by Phillips (1974) who distinguished between sensory storage and visual short-term memory, showing that the former could be masked by noise masks, but the latter was impervious to masking. Smithson and Mollon (2006) also concluded from their study that a mask cannot penetrate higher levels of visual analysis and leaves intact the conceptual, abstract representations of stimuli.

### Design and Procedure

The design and procedure remained the same as Experiment 2. The only change was a noise mask introduced immediately after the stimulus display for 250 ms (the same duration as the study display). Thereafter, the test display was immediately presented.

### Results

Repeated measures *ANOVA* showed the significant main effect of the mode of presentation, *F*(1,17) = 6.949, *MSE* = 0.260, *p* = 0.017, *partial η^2^* = 0.290, BF_01_ = 1.29, with simultaneous presentation being better than sequential presentation. The main effect of locations was also significant, *F*(1,17) = 43.690, *MSE* = 0.446, *p <* 0.001, *partial η^2^* = 0.720, BF_10_ = 4.25 × 10^6^, with performance being better with unchanged locations than random locations. The interaction effect was also significant, *F*(1, 17) = 5.468, *MSE* = 0.351, *p* = 0.032, *partial η^2^* = 0.243, BF_10_ = 2.80. The difference of unchanged and randomized location was higher in simultaneous [*t*(17) = 5.761, *p* = 0.001] than sequential [*t*(17) = 3.981, *p* = 0.001] presentation. Figure 2 shows the results. The similar pattern of results for Experiment 2 and 5 is clearly visible. The three-way *ANOVA* carried out to compare Experiment 2 and 5 showed that neither the main effect of experiments nor any of the interactions involving experiments were significant.

### Discussion

Visual noise masks were used in this experiment to eradicate the effect of iconic memory in the performance of the participants. It was of particular interest whether performance in the unchanged locations condition for simultaneously presented stimuli would reduce as compared to Experiment 2. However, there was simply no effect of the mask on the general performance level of the participants or particularly with simultaneous presentation and unchanged locations.

A three-way *ANOVA* was performed with Experiment 2, 3, 4, and 5 as the between-subjects factor and mode of presentation and locations as repeated measures. Neither the main effect of experiments nor any interaction of experiments with other factors was significant. Bayes factors were computed for every combination of main and interaction effects. A model comprising the three-way interaction and all the main and two-way interaction effects (BF_10_ = 2.026 × 10^27^) was compared with the model with only the main and two-way interaction effects (BF_10_ = 7.388 × 10^27^). The data fit better with the model without the three-way interaction by a factor of 3.64:1.

## General Discussion

This research aimed to compare sequentially presented feature bindings with simultaneously presented bindings, hoping to reveal the factors, which lead to differential performance with these two modes of presentation. Previous studies, which compare simultaneous and sequential presentations, have shown mixed results, although in most studies the performance of participants is better with simultaneous presentation. We particularly designed experiments to disentangle the confound of locations with simultaneous presentation as many theories and studies have stressed the importance of locations in the process of binding (e.g., Wolfe, 1994; Treisman and Zhang, 2006; Logie et al., 2011).

The results of Experiment 1 show that merely adding a temporal cue, i.e., presenting stimuli one by one to build up the study display has no differential effect on the performance of the participants as compared to when the stimuli are simultaneously presented. Nevertheless, locations had a significant effect, with performance being significantly better when locations remained the same, than when they were randomized from study to test. This was true regardless of whether the stimuli were presented simultaneously or sequentially.

However, Experiment 2 showed a significant difference between the two modes of presentation as well as a significant interaction. In this experiment, stimuli were presented in the sequential mode of presentation such that as one stimulus was presented, the previous one vanished. Performance was worse with sequential presentation as compared to simultaneous presentation perhaps because the participants were never able to “see” the stimuli in relation to each other in the sequential presentation condition. Presumably, they were building up a mental representation of the stimuli presented in sequence, as they knew they would be tested with a whole display, having understood the experimental task, and having done many practice trials. However, in building this mental pattern/representation, it was harder for them to take advantage of the spatial relationship among the stimuli with sequential presentation such that one stimulus vanished as the next was presented. In Experiment 1, where the study display was gradually built up, they could take advantage of unchanged locations and hence the performance is not any different in the sequential presentation condition as compared with the simultaneous presentation condition.

Coming back to Experiment 2, encoding the stimuli in a configuration or pattern led to enhanced performance in the simultaneous presentation condition with unchanged locations. However, unchanged locations from study to test did not confer any advantage if the stimuli were sequentially presented. Indeed, for sequentially presented stimuli, performance was statistically not different for unchanged and random locations, indicating that location was simply not a factor in the performance of the participants with sequentially presented stimuli.

These results are in contrast to that of Pertzov and Husain (2014) who used sequential presentations of four stimuli testing the binding between color and orientation, and compared performance in same and different locations. They showed that there were less errors in the “different locations” condition. However, the differences in their experimental task as compared to ours must be noted. In their experiment, same location condition meant presenting the stimuli in exactly the same location one after the other, which does not make location unnecessary to the task, rather it makes it relevant, and thus, a factor creating confusion. In contrast, in our experiments, the stimuli are presented in different locations, which remain unchanged from study to test. Thus, in our task, locations aid in differentiating the stimuli. In their “different” locations condition, the stimuli are presented in different locations in the sequence, and tested by a probe in the center of the screen. In this case too, locations are not irrelevant to the task and the binding of other features (color and orientation in this case) may be addressed through locations, as suggested by feature integration theory (Treisman and Sato, 1990) and related studies (e.g., Treisman and Zhang, 2006; Logie et al., 2011).

Could the relatively long presentation time of 1,000 ms for all four stimuli cause better performance because in sequential presentation only 250 ms was given for each stimulus? If this was so, then giving less time to perceive stimuli in the simultaneous condition should decrease performance. However, the results of Experiment 3 showed that this was not the case. Even reducing the presentation time of the simultaneously presented stimuli to 250 ms and thus making it equal to the presentation time of a single stimulus in the sequential condition did not affect the performance of the participants. Probably this is because all stimuli in the simultaneous presentation condition have already been encoded even at 250 ms and performance has therefore reached an asymptote. Vogel et al. (2006) have suggested that about 60 ms are required to encode the first stimulus, followed by 50 ms per stimuli for the rest of them. Although this study was with colored squares (uni-feature objects), in an earlier study, Luck and Vogel (1997) reported that the capacity of visual short-term memory is about the same for uni-feature and multi-feature objects, which is four objects. Despite suggestions that visual working memory capacity is also affected by complexity and resource demand of stimuli (Alvarez and Cavanagh, 2004; Ma et al., 2014), we believe that our four objects, which are rather simple conjunctions of color and shapes, are well within visual working memory capacity, and so presumably all stimuli in the display could be encoded within 250 ms.

Some researchers have argued that what happens in the maintenance period is as important as initial encoding; and performance is worse with sequential presentation because each stimulus gets overwritten by subsequent stimuli (Ricker and Cowan, 2014). The fourth experiment was designed to test whether introducing blank intervals after every stimulus would allow the participant to consolidate its memory and/or protect it from being overwritten by the next stimulus and hence increase the performance of the participants in the sequential presentation condition. The results did show no significant difference between sequential and simultaneous presentation conditions. However, a comparison of Experiment 2 and 4 revealed that performance did not increase in sequential presentation condition. Rather, it *decreased* in the simultaneous presentation condition with unchanged locations, probably because of the very long presentation time in this condition leading to forgetting. Thus, the blank intervals, which yielded better performance with sequential presentation in the experiment by Ricker and Cowan (2014) conferred no advantage in the sequential presentation condition in our experiment. This might be because the experiments by Ricker and Cowan were testing memory for unfamiliar shapes, whereas we were testing feature bindings. Presumably, feature bindings are already represented in the visual short-term memory beyond iconic memory, and hence do not benefit by the opportunity of consolidation (or protection) given by blank intervals to rather fragile representations of features in the initial stage of processing.

The idea that feature bindings are represented in visual short-term memory beyond iconic storage is also substantiated by Experiment 5, where we attempted to use pattern masks comprising visual noise to disrupt iconic memory representations. However, there were no significant differences in the performance of the participants as compared to Experiment 2, substantiating that feature bindings are held in the visual working memory and are thus only affected by factors, which organize information after basic perceptual processing. Supportive evidence that VSTM representations are immune to masking is offered by several studies (e.g., Phillips, 1974; Smithson and Mollon, 2006; Sligte et al., 2008).

Consequently, we conclude that the differences between simultaneous and sequential presentations are not due to ostensible perceptual differences, but due to factors and processes that affect the organization of material/stimuli in the visual working memory. All manipulations, which could have affected perceptual processing of stimuli, viz., altering the presentation time, and inserting blank intervals after each stimulus presented in a sequence, or presenting a noise mask after the stimulus presentation, had no effect on the levels of performance of the participants. So the differential performance between simultaneous and sequential modes of presentation cannot be attributed to factors in perceptual processing. The significant interaction effect obtained in all experiments where stimuli were presented in the sequential condition such that one stimulus vanished as the next appeared substantiates that location as a feature contributes to making performance better with simultaneous presentation. The significant advantage of unchanged locations as compared to randomized locations is clear in the simultaneous presentation condition in all experiments. It is clear that this advantage accrues only when stimuli can be encoded in relation to each other, being presented together in multiple locations.

However, in the case of stimuli presented sequentially, location is simply not relevant to performance as keeping it the same or randomizing it has no effect on the performance of the participants. Perhaps this is because these stimuli are already represented in visual working memory. This idea is further substantiated by the last three experiments, which show that the performance in the sequential presentation condition is immune to manipulations designed to alter the encoding of stimuli such as changes to presentation time, or inserting blank intervals, or using a noise mask immediately after stimulus presentation. Also, as suggested by one of the reviewers, performance in the sequential presentation condition could have been worse because participants were required to maintain items for a longer duration in this condition, particularly in the experiment where blank intervals were inserted. Clearly, this difficulty in “maintenance” would occur only if the stimuli were already present in visual working memory. In sum, we speculate that sequences are encoded or consolidated into visual working memory relatively automatically and perhaps sooner as compared to simultaneously presented stimuli. Analogous to the advantage that sentences have over lists in verbal working memory due to long-term knowledge (Allen et al., 2018), perhaps sequences of visual stimuli too benefit from temporal cues which are simply absent in simultaneously presented stimuli. Alternatively, competition among simultaneously presented stimuli may act as a bottleneck and retard the progress of these early visual representations into working memory. This idea is supported by the experimental finding that differences between simultaneous and sequential presentations are evident only at larger set sizes and are not shown with set sizes within working memory capacity (Igel and Harvey, 1991; Dent and Smyth, 2006). Another explanation could be that participants are using different strategies to process simultaneously and sequentially presented stimuli. Udale et al. (2018) have argued that participants can use different strategies to encode and process stimuli when required by task demands in the absence of locations being relevant. In fact, they also suggest individual differences among participants in the use of these strategies. Much further research is required to explore exactly which factors and processes in visual working memory are relevant for binding sequentially presented stimuli.

On the basis of current studies, it may be concluded that while performance with simultaneous presentation relies on location information, performance with sequential presentation is relatively immune to presence/absence of location information. It is also clear that post-perceptual processes within visual working memory are presumably responsible for the differences in performance due to simultaneous and sequential presentation.

## Data Availability Statement

The datasets generated for this study are available on request to the corresponding author.

## Ethics Statement

The studies involving human participants were reviewed and approved by Research Ethics Committee, Department of Psychology, Chaudhary Charan Singh University, Meerut. The participants provided their written informed consent to participate in this study.

## Author’s Note

AB carried out this research as part of PhD. He was supported for PhD by a fellowship from the Ministry of Human Resource Development, India.

## Author Contributions

AB, SY, and SJ together conceptualized the study and wrote the paper.

### Conflict of Interest

The authors declare that the research was conducted in the absence of any commercial or financial relationships that could be construed as a potential conflict of interest.
